# High prevalence of Seoul hantavirus in a breeding colony of pet rats

**DOI:** 10.1017/S0950268817001819

**Published:** 2017-10-02

**Authors:** L. M. McELHINNEY, D. A. MARSTON, K. C. POUNDER, H. GOHARRIZ, E. L. WISE, J. VERNER-CARLSSON, D. JENNINGS, N. JOHNSON, A. CIVELLO, A. NUNEZ, T. BROOKS, A. C. BREED, J. LAWES, Å. LUNDKVIST, C. A. FEATHERSTONE, A. R. FOOKS

**Affiliations:** 1 Department of Virology, Animal and Plant Health Agency (APHA), Weybridge, Surrey, UK; 2 HPRU Emerging and Zoonotic Infections, Institute of Infection and Global Health, University of Liverpool, Liverpool, UK; 3 Department of Medical Biochemistry and Microbiology and Department of Medical Sciences, Zoonosis Science Centre, Uppsala University, Uppsala, Sweden; 4 Department of Pathology, APHA, Weybridge, Surrey, UK; 5 Rare and Imported Pathogens Laboratory, Public Health England, Porton Down, Salisbury, UK; 6 Department of Epidemiological Sciences, APHA, Weybridge, Surrey, UK; 7 Epidemiology and One Health Section, Department of Agriculture and Water Resources, Canberra, Australia; 8 Laboratory of Clinical Microbiology, Uppsala University Hospital, Uppsala, Sweden; 9 APHA, Thirsk Veterinary Investigation Centre, Thirsk, UK

**Keywords:** Hantavirus, Rabies (animal), Rabies (human), Virology (human) and epidemiology, Zoonoses

## Abstract

As part of further investigations into three linked haemorrhagic fever with renal syndrome (HFRS) cases in Wales and England, 21 rats from a breeding colony in Cherwell, and three rats from a household in Cheltenham were screened for hantavirus. Hantavirus RNA was detected in either the lungs and/or kidney of 17/21 (81%) of the Cherwell rats tested, higher than previously detected by blood testing alone (7/21, 33%), and in the kidneys of all three Cheltenham rats. The partial L gene sequences obtained from 10 of the Cherwell rats and the three Cheltenham rats were identical to each other and the previously reported UK Cherwell strain. Seoul hantavirus (SEOV) RNA was detected in the heart, kidney, lung, salivary gland and spleen (but not in the liver) of an individual rat from the Cherwell colony suspected of being the source of SEOV. Serum from 20/20 of the Cherwell rats and two associated HFRS cases had high levels of SEOV-specific antibodies (by virus neutralisation). The high prevalence of SEOV in both sites and the moderately severe disease in the pet rat owners suggest that SEOV in pet rats poses a greater public health risk than previously considered.

## INTRODUCTION

Hantaviruses (order *Bunyavirales*, family *Hantaviridae*, genus *Orthohantavirus*) are single-stranded RNA viruses. Unlike other members of the *Bunyavirales*, hantaviruses are not transmitted by arthropods but primarily by rodents of the families *Cricetidae* and *Muridae* [1]. Each hantavirus appears to be adapted and largely restricted to an individual reservoir host species. However, evolutionary analyses support both host–virus co-divergence and cross-species transmission, with Chiroptera (bat) or Soricomorpha (mole and shrew) hantaviruses emerging before the rodent hantaviruses [2].

At least six rodent-borne hantaviruses have been reported in Europe; *Dobrava-Belgrade hantavirus* (DOBV), *Saaremaa hantavirus* (since proposed as a sub-species of DOBV), *Seoul hantavirus* (SEOV), *Puumala hantavirus* (PUUV), *Tatenale hantavirus* (TATV) and *Tula hantavirus* [1, 3]. The most common and widespread hantavirus across Europe is PUUV, which is associated with the mildest form of haemorrhagic fever with renal syndrome (HFRS) [1]. In the UK, only SEOV and TATV have been detected in wild rodents; in Brown rats (*Rattus norvegicus*) and a field vole (*Microtus agrestis*), respectively [3, 4].

*Rattus norvegicus* (Norway or Brown rats, subsequently referred to here as rats) are known to act as a reservoir and vector of several zoonotic pathogens [5, 6] and are considered responsible for the global dissemination of SEOV hantavirus [7]. SEOV causes mild-to-severe HFRS in humans [8], whilst the zoonotic potential of the field vole borne TATV has yet to be determined.

Hantavirus transmission to humans is primarily via inhalation of aerosolised virus in contaminated rodent urine and faeces. Whilst infected reservoir hosts are asymptomatic, human infections are thought to lead to two clinical manifestations, HFRS and hantavirus cardiopulmonary syndrome (HCPS), with varying degrees of morbidity and mortality [1].

Historically in Europe, relatively few human SEOV-associated HFRS cases were reported. Serologically confirmed SEOV cases were linked to infected laboratory rats [9] or proposed to have arisen from exposure to infected wild rats (online Supplementary Table S1). In addition, 16 serologically confirmed SEOV-associated HFRS cases were identified retrospectively in patients hospitalised in Northern Ireland between 1989 and 1992 with fever, acute kidney injury (AKI) and thrombocytopenia [10]. More recently, there has been a confirmed HFRS case in Lyon, France [11] and a growing number of HFRS cases in the UK likely to be associated with wild and captive rats [4, 12, 13]. SEOV hantaviral RNA was also detected in two of 20 pet rats imported into Sweden from the UK in 2011 [14]. SEOV-specific antibodies or viral RNA have been detected in wild rats in the UK [4, 15, 16], Belgium [17–19], France [20, 21], Portugal [22] and The Netherlands [23]. Antibodies to Hantaan virus (HTNV) were reported in the UK in healthy (9·6%) and chronically ill (23%) cats [24], but due to cross-reactivity amongst *Murinae*-associated hantaviruses, this may have indicated exposure to SEOV or other hantaviruses rather than HTNV. However, it is thought that cats and dogs do not play a role in the maintenance and transmission of hantaviruses and most likely represent dead end hosts [25].

In 2013, following a serologically confirmed HFRS case in North Wales (online Supplementary Table S1), SEOV RNA was detected in two pet rats and identified as the likely source of infection in the owner [8]. The two rats were originally purchased from a breeding colony in Cherwell and preliminary screening of blood sampled from the group detected hantavirus RNA in 7/21 rats [12]. In response to this, a further investigation into the colony was undertaken. The colony was voluntarily culled and organ samples screened for hantavirus. In addition, three rats were also investigated from a household in Cheltenham, Gloucestershire, where a mother and daughter developed HFRS in 2013. One of the rats from this location had been purchased from the Cherwell breeding colony in 2012. The purpose of this investigation is to confirm the prevalence of SEOV hantavirus in pet rats and provide further epidemiological evidence for pet rats as the source of HFRS in humans.

## METHODS

### Cherwell colony

The owner's consent was obtained to humanely euthanase the pet rats in their breeding colony in Cherwell, Oxfordshire. In February 2013, 20 rats were killed and one rat was found dead. Lung and kidney material were removed from all animals for hantaviral RNA screening and the carcases were stored at −80 °C. Heart, liver, salivary gland and spleen were also removed from one individual (#3784), a male rat introduced into the Cherwell colony, possibly from Germany, a few months before the owner was hospitalised with HFRS in November 2011 (Table 1). Additional organs were also removed from selected rats for histopathological investigation.

**Table 1.** Hantavirus nRT–PCR (L gene) and serology results for the 21 pet rats (Rattus norvegicus) in the Cherwell Colony

APHA ID	Colour	Gender	Hantavirus nRT-PCR		PCR status	SEOV ELISA (adjusted OD)	SEOV FRNT	Serology status	PM observations/notes
			Lung^a^	Kidney^a^					
3776	Brown	Female	Neg (0/4)	Neg (0/4)	Neg	0·396	1 : 160	Pos	Healthy
3777	Brown	Female	Pos (2/3)	Pos	Pos	0·2	1 : 2560	Pos	Healthy
3778	Brown	Female	Pos (1/4)	Neg (0/4)	Pos	No blood	No blood	nd	Found dead. Vaginal discharge, nose bleed
3779	Brown	Female	Neg (0/4)	Pos (4/4)	Pos	0·517	1 : 160	Pos	Fatty liver
3780	Brown	Female	Pos	Pos	Pos	nd	⩾1 : 40	Pos	Healthy
3781	Brown/white	Female	Pos	Pos	Pos	0·79	>1 : 10 240	Pos	Healthy
3782	Brown/white	Female	Pos	Pos	Pos	0·643	>1 : 10 240	Pos	Healthy
3783	Brown	Female	Neg (0/4)	Pos (4/4)	Pos	0·76	1 : 640	Pos	Healthy
3784	Brown	Male	Pos	Pos	Pos	1·05	1 : 10 240	Pos	Enlarged pale kidneys. Imported to UK in 2011.
3785	Brown	Female	Pos	Pos	Pos	0·606	1 : 2560	Pos	Healthy
3786	Black/white	Female	Neg (0/4)	Neg (0/4)	Neg	0·686	>1 : 2560	Pos	Healthy
3787	Brown	Male	Pos	Pos	Pos	0·09	1 : 640	Pos	Healthy
3788	White	Female	Neg (0/4)	Neg (0/4)	Neg	0·245	1 : 640	Pos	Healthy
3789	Brown	Female	Pos	Pos	Pos	0·96	1 : 2560	Pos	1·5 cm tumour, hard mass in right mammary gland
3790	Brown	Male	Pos	Pos	Pos	0·676	1 : 640	Pos	Healthy
3791	Golden	Male	Neg (0/4)	Pos	Pos	0·89	1 : 2560	Pos	Healthy
3792	Dark brown	Female	Pos	Pos	Pos	0·63	>1 : 10 240	Pos	Healthy
3793	Pale Brown	Male	Pos (1/3)	Pos	Pos	0·33	1 : 2560	Pos	Healthy
3794	White	Female	Neg (0/4)	Neg (0/4)	Neg	0·875	1 : 160	Pos	Healthy
3795	Mix colour	Male	Pos	Pos	Pos	0·633	>1 : 10 240	Pos	Healthy
3796	Brown/white	Female	Pos (4/4)	Neg (0/4)	Pos	0·461	1 : 640	Pos	Healthy
Total positive			14/21	15/21	17/21			20/20	

OD, optical density; Pos., positive; Neg., negative; nd, not determined.

a Unless otherwise stated, the reported RT–PCR results were observed in triplicate. Positive controls: D+VE RAT (ELISA 1·0 and FRNT 1 : 2560) and KAP KIN (ELISA 0·624 and FRNT 1 : 640).

### Cheltenham household

The three rats from the Cheltenham household were submitted separately with the owner's consent between 2014 and 2016. Kidney samples were screened for hantavirus RNA.

### Screening for hantavirus RNA

Approximately 50–100 mg of lung or kidney tissue was homogenised in 1 ml TRIzol^®^ Reagent (Invitrogen, Life Technologies, Paisley, UK). RNA was extracted from the homogenate according to the manufacturer's instructions (Invitrogen, Life Technologies, Paisley, UK). The RNA samples were reverse transcribed using random hexamers and screened for hantavirus as previously described [3] employing a pan-hantavirus nested RT–PCR directed against partial polymerase (L) gene sequences [26].

### *Cytochrome b* (Cyt b) PCR

Morphological species determination of small mammals was confirmed by molecular identification using degenerate *cyt b* primers [27].

### Phylogenetic analysis

The positive amplicons were sequenced using a BigDye Terminator 3·1v Cycle Sequencing Kit on an ABI3130xl genetic analyser (Applied Biosystems/Life Technologies, Paisley, UK). Sequence alignments and maximum likelihood phylogenetic trees were generated in MEGA6·06. A Tamura three-parameter nucleotide substitution model with *γ* rate variation was determined to best fit the data using Akaike Information Criterion (AIC) in MEGA 6·0 [28], with bootstrap replications of 10 000 [29].

### Serology

Twenty serum samples obtained from the Cherwell rats (one rat was found dead and blood was unavailable) were screened using the Focus Reduction Neutralisation Test (FRNT), the gold standard assay for typing hantavirus antibody responses. An 80% reduction of the number of foci, as compared with the virus control, was used as a criterion for virus neutralisation titres and the titrations were performed as described earlier [30] (online Supplementary Material). Serum samples collected in January 2013 from two HFRS patients (Cherwell and Wrexham) were also tested using specific hantavirus FRNTs against SEOV, DOBV, PUUV and HTNV.

Sera (two human and 19 rats) were also screened using an in house SEOV ELISA as described previously [23].

### Histopathology

Gross post-mortem examination was performed on rat carcasses. Tissue samples were collected and fixed in 10% neutral buffered formalin for 1 week, then processed to paraffin wax blocks. Four micrometre-thick sections were cut and stained with haematoxylin and eosin for histopathological examination. Lung and kidney tissues were examined from all 21 rats. In addition, heart, spleen, brain and salivary gland from 11 rats, liver from eight rats, pancreas from three rats, and single samples of duodenum, mandibular lymph node and a skin mass were examined.

## RESULTS

### Cherwell rat breeding colony

Twenty-one rat carcasses were submitted from the Cherwell colony representing the total population of rats at the site. Kidney and lung samples were removed at necropsy and hantavirus RNA was detected in 17 of 21 (81%) rats in either the lung and/or kidney tissues; with 14/21 lung positive, 15/21 kidney positive and 12/21 in both (Table 1). SEOV RNA was more reliably detected in the kidney than the lung tissue in replicate testing (Table 1). For the single imported rat (#3784), SEOV RNA was detected in the heart, kidney, lung, salivary gland and spleen but not in the liver. Infection prevalence was higher in males 100·0% (95% CI 54·1–100·0%) than females 73·3% (95% CI 44·9–92·2%), although not statistically significant (Fisher's exact test, *P* = 0·2807).

### Cheltenham household

In February 2013, an adult female who had presented with viral meningitis and subsequent AKI volunteered herself for hantavirus serological testing as an owner of pet rats. In March 2013, she was found to be seropositive to hantavirus and the risk to her family from her rats was discussed with her. In August 2013, her 11-year-old daughter was admitted to hospital with headache, malaise, anorexia, acute pyrexia and an acute kidney injury, which resolved without needing dialysis. Laboratory tests indicated haematuria, elevated transaminases and thrombocytopenia and serologically confirmed HFRS. In addition, SEOV RNA was detected in her blood by nested RT–PCR [26] with 100% sequence identity (partial L segment) to Cherwell SEOV.

The Cheltenham household had purchased a rat in November 2012 from the Cherwell breeding colony. The rat had eight pups, three were kept at the residence and five returned to Cherwell. In late June 2013, the owner was asked to take part in a television documentary about pet rats. Four rats from two different breeding colonies were obtained for this documentary: two adult females from the Cherwell colony, and two baby rats from a breeding colony in Gloucester. The owner of the Gloucester colony, which at one point consisted of up to 150 rats, was seropositive to hantavirus, but had not reported clinical disease. There was a regular exchange of rats between the Gloucester and Cherwell colonies. Hantavirus RNA was detected in the kidney tissue from all three rats submitted from Cheltenham.

### Phylogenetic analysis

#### Hantavirus

**Fig. 1.**
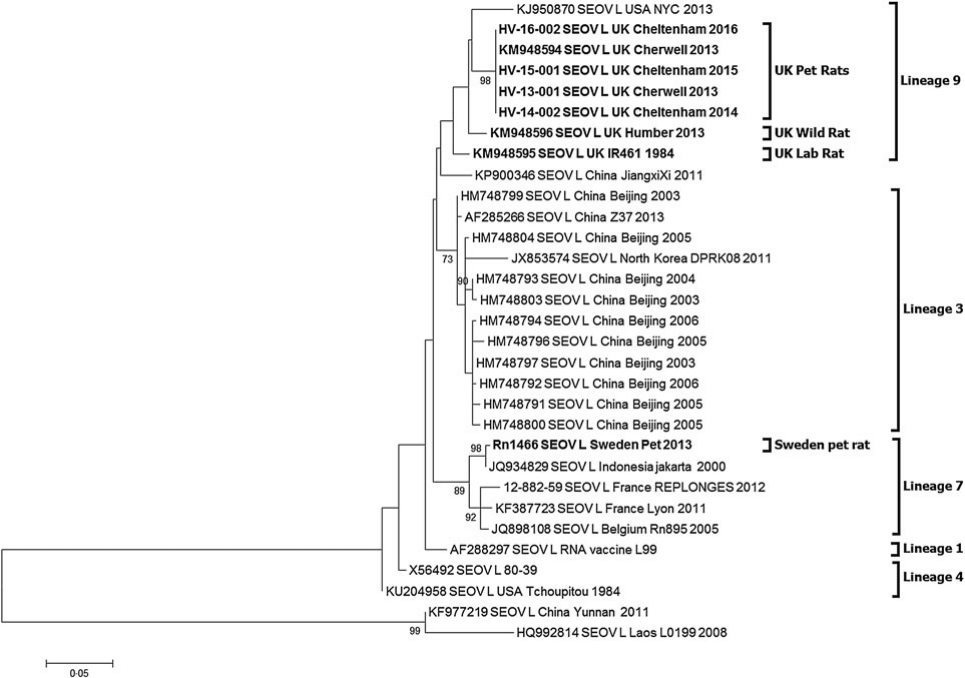
Maximum likelihood phylogenetic tree for SEOV partial L segment (333nt) sequences (*n* = 31) using model Tamura three-parameter model with *γ* distribution in the MEGA6 package of software with bootstrap of 10 000 [28, 29]. The trees are drawn to scale, with branch lengths measured in the number of substitutions per site. The scale bar indicates amino acid substitutions per site. Only bootstrap support of >70% are shown. The phylogenetic positions of the UK pet rats are shown in relation to representative Seoul virus strains. Genbank accession numbers are shown next to taxa names.

Partial hantavirus L segment sequence (333nt) was obtained for 10 of the 17 SEOV-positive pet rat strains and the three Cheltenham SEOV positive pet rat strains. BLAST search confirmed that the sequence was SEOV. All 13 SEOV sequences were 100% identical to each other (Accession KY688127-KY688130) and the previously published Cherwell strain (Accession KM948594). The sequences shared 97% identity to the UK wild rat SEOV Humber strain (KM948596) in this genetic region and 96% to the UK laboratory strain IR461 (KM948595). Phylogenetic analysis of the SEOV-positive pet rats (Fig. 1) shows them clustering with the previously published UK SEOV strains; laboratory rat SEOV strain IR461 and wild rat Humber SEOV strain, located within the SEOV Phylogroup A lineage 9 [7, 19]. The UK SEOV strains clustered in lineage 9 with the Baxter SEOV strain (95% sequence identity), originating from a wild brown rat trapped in New York City in 2013 [5].

The SEOV/Sweden/RN1466/2013 sequence (Accession KY688131) obtained from the pet rat imported from the UK into Sweden in 2011 [14] clusters with SEOV lineage 7 rather than the lineage 9.

Excluding the diverse 2011 human SEOV (DLR2) from Yunnan, China (KF977219) and the 2008 *Rattus tanezumi* SEOV (L0199) from Laos (HQ992814), the remaining globally distributed SEOV sequences demonstrated between 91·6 and 100% sequence identity in the partial L segment.

#### Cyt b PCR

Analysis of partial *cyt b* gene sequences (833 bp) have proven useful in detecting host/virus relationships [7, 27], and thus were studied to assess the level of connectivity within and between wild and captive rats. *Cyt b* sequences were recovered from 18 of the 21 Cherwell pet rat samples; 16 samples from infected rats and two samples from non-infected rats. Seven variable sites were located within this partial sequence. No clear patterns of connectivity were identified as the UK pet rat and wild rat partial cyt b sequences assembled indiscriminately amongst global rat sequences (online Supplementary Fig. S1), and thus no discrete relationships could be determined.

### Serology

Twenty rats from Cherwell were bled and screened by SEOV FRNT and an in house SEOV ELISA (Table 1). All were seropositive by FRNT with SEOV-specific antibody titres >1 : 40. Some rats had very high titres, including the imported rat #3784 with a SEOV neutralising antibody titre of 1 : 10 240. Nineteen of the 20 rat sera were positive by the in-house SEOV ELISA (OD cut-off = 0·1) and #3787 was borderline at 0·09.

Serum from the two HFRS cases associated with the Cherwell rats were confirmed to contain specific antibodies to SEOV by FRNT virus neutralisation (Table 2). Case 1 (Wrexham, North Wales, male, 28 years old) was hospitalised with AKI and multi-organ failure in October 2012 and required ventilatory support and renal replacement therapy [8]. Blood collected in Janury 2013 had a specific SEOV antibody titre of 1 : 640. Case 2 (Cherwell, Oxfordshire, male), identified retrospectively, was hospitalised in November 2011 with fever, thrombocytopenia, splenomegaly and AKI. Blood collected over a year after hospitalisation (January 2013) from case 2 had a very high SEOV antibody titre of 1 : 10 240. Cross-neutralisation titres were detected against PUUV, DOBV and HTNV but considerably higher neutralising antibody titres were observed against SEOV, confirming the two patients had SEOV-associated HFRS (Table 2). The two human sera were strongly positive in the in house SEOV ELISA with OD values >1 (OD cut-off = 0·1).

**Table 2.** Hantavirus-specific virus neutralisation tests on human serum collected from the two HFRS cases (Wrexham and Cherwell)

UK human sera	SEOV ELISA (adjusted OD)	FRNT SEOV	FRNT PUUV	FRNT DOBV	FRNT HTNV
Case 1 Wrexham	1·386	1 : 640	1 : 40	1 : 160	1 : 40
Case 2 Cherwell	1·462	>1 : 10 240	1 : 40	1 : 640	1 : 160

### Histopathology

In four of the 21 rats, the kidneys exhibited chronic mild thickening of the Bowman's capsule, tubular ectasia with proteinosis (presence of eosinophilic material in the tubules) and epithelial attenuation, and lymphoplasmacytic interstitial nephritis. In one of the rats that had pale kidneys at gross post-mortem (#3784), similar renal changes were moderate in severity and accompanied by interstitial fibrosis. In 16 of the 21 rats, the lungs exhibited mild to marked chronic hyperplasia of bronchial-associated lymphoid tissue, periairway and perivascular lymphoplasmacytic infiltrates and occasional neutrophilic bronchopneumonia with bronchiectasis. In one rat (#3789), there was a mammary fibroadenoma (a benign mammary tumour), and in the rat that was found dead (#3778), there was a mild lipid hepatopathy that is presumed to have been caused by anorexia before death. Individual histopathological findings are summarised in online Supplementary Table S2.

## DISCUSSION

Domesticated rats are not generally considered a public health risk. However, this study shows a high prevalence (81%, 17/21) of SEOV-infected rats within a breeding colony. If these 21 rats are considered representative of the larger pet rat population, the prevalence for SEOV infection would be expected to be between 58·1–94·6% (95% binomial confidence interval). Of the four human contacts connected to the Wrexham and Cherwell sites, three had been exposed to SEOV (seropositive) of which two had moderately severe HFRS [8]. In addition, the SEOV-associated HFRS cases in the Cheltenham household and UK animal facility [9] were also moderately severe and required hospitalisation. The UK case histories support the possibility that SEOV HFRS can present as a broad spectrum of pathologies, from an apparent sub-clinical infection to multi-organ failure or death.

Diagnostically, SEOV RNA was most reliably detected in the kidney samples of rats with agreement for all replicate testing (either three or four tests per sample). The results also suggest that if lung tissue is used for diagnosis then replicate testing should be undertaken to ensure confidence in any negative results. Multiple organs were screened for the imported rat (#3784). SEOV RNA was detected in 5/6 organs tested, including the salivary glands which have previously been shown to be a source of direct transmission between rats during aggressive encounters [31]. The liver did not yield viral RNA in this individual despite multiple attempts. The liver was checked for inhibitors that could have affected the PCR. However, detection of the *cyt b* gene confirmed this not to be the case. Hence, the liver may not be a reliable diagnostic target in the absence of other tissue specimens.

Despite their disparate geographical separation, most SEOV variants published to date are genetically homogenous making it difficult to determine the precise source of introduction. However, phylogenetic analysis of the partial L sequence (333 nt) confirmed that there is geographical clustering of the UK SEOV sequences alongside the US Baxter SEOV strain within SEOV lineage 9. Such geographical clustering has been observed in China [7, 32] and France [21].

The partial L gene sequences for the Wrexham (Cherwell strain, KM948594), Cherwell colony and Cheltenham rats are 100% identical (Fig. 1) demonstrating a viral homogeneity not observed for other hantaviruses, and rare for RNA viruses in general, even within the relatively well-conserved polymerase gene. This is consistent with the emergence of the virus in three locations over a short time span (2011–2013) resulting from translocation of infected rats between the Cherwell colony and locations in Wales and Gloucestershire. The clustering of viral sequences from the UK wild rat, laboratory rats and pet rats may be suggestive of a single ancestor, as previously proposed in investigations of the laboratory rat cases [33]. Pet rats in the UK may have been recently exposed to wild rats and/or their excreta and then once infected with SEOV maintain the virus within the pet rat community. In this particular case, the original source of infection could have been the importation of rat #3784 to the UK in 2011, possibly from a breeding source in Germany. However, no German SEOV cases have been reported, so no sequences are available to corroborate this. In addition, the higher similarity of the #3784 SEOV strain (Cherwell) to the UK wild rat strain (Humber), compared with continental European wild rat SEOV sequences (France, Belgium), makes this hypothesis less likely. Alternatively, SEOV may have been present in the pet rat population for a prolonged period but now there are increasing opportunities for dissemination (rat exhibitions, rat owner networks, sales). The sub-clinical disease in rats and the non-pathognomonic nature of SEOV infections in humans have probably supported under-reporting and misdiagnosis. Prior to routine screening and enhanced biosecurity measures, laboratory rats may have historically been infected prior to domestication or subsequently via wild rats. However, there is a paucity of data on SEOV phylogeny and the sequence homogeneity reported [19, 21, 32], which may obscure the current epidemiological picture.

Subsequent to the 1994 report by McKenna *et al*. [10], the limited surveillance in Europe to date has likely failed to reveal the true prevalence of SEOV. It is not known to what extent the virus is localised or maintained within wild rat populations following its introduction to a naïve population. The detection of the SEOV Humber strain in 2/4 (50%) trapped rats on the farm of the HFRS patient in Yorkshire [4] compares to earlier UK wild rat seroprevalence estimates of 21·6% (Northern Ireland *n* = 51) [16] and 4% (England *n* = 100) [15]. No SEOV RNA was detected in the lungs of 133 wild rats trapped in the port city of Liverpool, UK [3], whereas SEOV RNA was detected in the lungs of 18/128 (14%) wild rats trapped in and around the port city of Lyon, France [21]. In the Netherlands, 3/16 (19%) rats were recently shown to have SEOV-specific neutralising antibodies in a region where earlier attempts to confirm SEOV seropositivity by FRNT in 161 brown rats had failed [23]. Detection of the pathogen or the presence of specific neutralising antibodies (FRNT) unambiguously confirms that SEOV is present in localised foci in European brown rats. However, it is not yet clear whether the increased detection of SEOV in Europe is suggestive of an emerging zoonotic pathogen or rather enhanced surveillance and clinical awareness. Earlier studies and serologically confirmed HFRS cases in the UK (online Supplementary Table S1) suggest the latter may be more probable.

The SEOV partial L gene sequence (Accession KY688131) obtained for the pet rat imported from the UK into Sweden [14] was more similar to the geographically diverse lineage 7 (that includes SEOV sequences from France, Belgium, Indonesia, Vietnam, Singapore and Cambodia) than to the UK SEOV strains of lineage 9. It is unclear if the pet rat became infected in Sweden or the UK [14]. If the latter is the case, then additional as yet undetected strains of SEOV may be circulating in the UK.

Microbiologists are now encouraged to view pathogen epidemiology more holistically and seek to identify host markers, which may reflect the pathogen evolution and phylogeography. Previous reports of hantavirus phylogeny have included the use of cyt b sequences to identify host–pathogen relationships [7, 27]. However, in this study, the *cyt b* gene sequences for both the pet and wild rats are apparently randomly located on the phylogenetic tree (online Supplementary Fig. S1) with no apparent relationships to each other. A similar lack of connectivity can be observed for the cyt b sequences obtained for a selection of 17 wild rats trapped in and around Lyon, France [21]. This suggests that whilst the *cyt b* gene is useful for confirming the host species, it may not be the optimum host marker for determining finite intra-species relationships or population connectivity nor to identify the geographic origin of an imported or introduced rat. With host genetic data becoming more accessible to microbiologists with the increasingly routine application of next-generation sequencing technology, other approaches may provide greater resolution [34].

In humans, hantaviruses can induce vascular disease seen clinically as HFRS and HCPS [1] with overlap between these two syndromes [35]. On the other hand, rats and other natural rodent hosts are asymptomatic carriers of hantavirus, and pathological lesions have not been reported, despite widespread distribution of antigen in tissues [36]. Histopathological examination of the tissues from rats in this outbreak revealed common incidental lesions only. Lungs from 16 of the 21 rats had chronic pneumonia ranging from mild hyperplasia of bronchial-associated lymphoid tissue to suppurative bronchopneumonia with bronchiectasis. Similar lesions are common in domestic rats and are associated with pathogens that are widespread, predominantly *Mycoplasma pulmonis* or cilia-associated respiratory bacillus [37]. Kidneys from four of the 21 rats had changes that were consistent with chronic progressive nephropathy (CPN), including Bowman's capsule thickening, interstitial nephritis and tubular degeneration. CPN is a common progressive disease seen in older rats. The cause of CPN is unknown, but it has not been linked to viral infections [36]. Although being confirmed as being hantavirus carriers by PCR and serology, these rats failed to show specific pathological lesions.

This study confirms the high prevalence of SEOV in domestic rats associated with HFRS cases in those in close contact with them. This study also reports the regular movement of rats between owners. In 2014, the Pet Food Manufacturer's Association estimated there to be 100 000 pet rats within 28 000 households in the UK and a large network of pet rat owners (National Fancy Rat Society) exists that supports the complex mixing of pet rats for breeding, pet sitting and exhibitions. Such interactions may have facilitated widespread virus dissemination throughout the pet rat community. Indeed in one study, 26/79 (32·9%) UK pet rat owners were seropositive to hantavirus compared with 10/300 (3·3%) of healthy blood donors (Public Health England, unpublished data). Such widespread interactions, combined with the genetic homogeneity of the SEOV strains, make tracing the original source of SEOV infection in an outbreak extremely challenging.

The domestication of rats has long been known to influence their behaviour, morphology and physiology compared with their wild ancestors. Whilst domesticated rats retain the ability to demonstrate aggressive behaviour when provoked, their threshold of provocation and thus frequency of aggression is significantly diminished compared with those in the wild [38, 39] and may enhance the passive spread of virus within a colony. Whilst SEOV transmission is believed to be high amongst aggressive breeding males [40], wild rats are less likely to form social hierarchies, instead maintaining greater spatial distances from conspecifics and a more heightened sense of fear to humans than domesticated rats [39]. Such behavioural differences may influence disease transmission and support the different seroprevalence rates observed between the captive colony of rats in this study (100%) and the free roaming wild rats elsewhere.

It is possible that the high levels of neutralising antibodies (1 : 10 240) in the Cherwell HFRS case (case 2), 14 months after infection, may indicate subsequent immune stimulation from his continued exposure to SEOV-infected pet rats and excreta or viral persistence. However, high levels of neutralising antibodies have previously been observed in the convalescent sera of patients several decades after infection with hantaviruses without subsequent exposure and thus the high antibody levels detected in case 2 are more likely to be associated with the individual host's immunological response and the characteristics of the responsible hantavirus [41–43]. The high prevalence of SEOV in both the Cherwell breeding colony and Cheltenham colony and the severity of disease in the pet rat owners is most likely to have resulted from the greater and more sustained level of exposure to SEOV through rat mixing and husbandry practices, than previously acknowledged with wild rat-associated HFRS cases.

## Supplementary material

For supplementary material accompanying this paper visit https://doi.org/10.1017/S0950268817001819. click here to view supplementary material
