# Supplementary material for: High prevalence of Seoul hantavirus in a breeding colony of pet rats
Source: Epidemiol Infect. 2017 Oct 2;145(15):3115–24. doi: 10.1017/S0950268817001819 (PMC9148732; doi:10.1017/S0950268817001819)
Supplement: Supplementary file 1 [file S0950268817001819sup001.doc]

**Supplementary Material**

**Title: High Prevalence of Seoul hantavirus in a Breeding Colony of Pet Rats**

McElhinney, L.M.^1,2^, Marston, D.A.^1^, Pounder, K.C.^2^, Goharriz, H.^1^, Wise, E.L.^1^, Verner-Carlsson, J.^3^, Jennings, D.^1^, Johnson, N.^1^, Civello, A.^4^, Nunez, A.^4^, Brooks, T.^2,5^, Breed, A.C. ^6^, Lawes, J.^6^, Lundkvist, Å.^3^, Featherstone, C.A.^7^ and A.R. Fooks^1,2^

^1^Animal and Plant Health Agency (APHA), Weybridge, Surrey, UK; ^2^HPRU Emerging and Zoonotic Infection, Institute of Infection and Global Health, University of Liverpool, Liverpool, UK; ^3^Department of Medical Biochemistry and Microbiology and Department of Medical Sciences, Zoonosis Science Centre, Uppsala University, and Laboratory of Clinical Microbiology, Uppsala University Hospital, Uppsala, Sweden; ^4^ Pathology, APHA, Weybridge, Surrey, UK; ^5^ Rare and Imported Pathogens Laboratory, Public Health England, Porton Down, Salisbury, UK; ^6^ Epidemiology, APHA, Weybridge, Surrey, UK; ^7^APHA, Thirsk Veterinary Investigation Centre, Thirsk, UK

**Corresponding author:** Dr Lorraine McElhinney; lorraine.mcelhinney@apha.gsi.gov.uk

**Supplementary Table 1** Possible SEOV associated HFRS cases in the UK

| **Year** | **Sex (age)** | **Location** | **Sero +ve** | **Clinical Disease?** | **Likely exposure source** | Reference |
| --- | --- | --- | --- | --- | --- | --- |
| 1977 | Various | Sutton | 10 | 4 | Laboratory rats | Lloyd & Jones, 1986 |
| 1983 | Male (21yrs) | Glasgow | 1 | 1 | Unknown | Walker *et al.*, 1985 |
| 1988 | Male (18 yrs) | Glasgow | 1 | 1 | Boating pond attendant, wild rats | Kudesia *et al.*, 1988 |
| 1989-1992 | Various | Northern Ireland | 16 | 16 | Wild rats | McKenna et al., 1994 |
| 1991 | Male (21yrs) | Somerset | 1 | 1 | Garden Centre Supervisor, wild rats | Pether & Lloyd, 1993 |
| 1991 | Male (42yrs) | Somerset | 4 | 1 | Local waterways? Wild rodents? | Pether *et al.*, 1991 |
| 1991 | Female (64yrs) | Somerset | 1 | 1 | Local waterways? Wild rodents? | Phillips *et al.*, 1991 |
| 1991 | Female (16yrs) | Sheffield | 2 | 1 | Poultry farm visit | Rice *et al.*, 1993 |
| 1991 | Female (18yrs) | Sheffield | 1 | 1 | Rat infestation | Rice *et al.*, 1993 |
| 1991-93 | Various | Hereford/Preston | 41 | 0 | Farmers, wild rodents | Coleman, 2000 |
| 1992 | Various | Somerset | 27 | 26 | Farmers, sewage workers, wild rats | Pether & Lloyd, 1993 |
| 1994 | Male (10yrs) | Nottingham | 1 | 1 | Recent rat infestation | Watson *et al.*, 1997 |
| 2009 | ? | Yorkshire & Humber | 1 | 1 | Recent rat infestation | Jameson *et al.*, 2013a |
| 2011 | Male (?yrs) | Yorkshire & Humber | 1 | 1 | Recent rat infestation | Jameson *et al.*, 2013a |
| 2011 | Male & Female | Oxfordshire | 2 | 1 | Pet rats | Jameson *et al.*, 2013b, This study |
| 2012 | Male (28yrs) | Wrexham | 1 | 1 | Pet rats | Jameson *et al.*, 2013b, This study |
| 2013 | 2 Females (mother & daughter) | Gloucestershire | 3 | 2 | Pet rats | This study |
| 2015 | Male (?yrs) | Glasgow | 1 | 1 | Pet rats | unpublished |
| 2015 | 3 Males (23, 26 & 56 yrs) | Cardiff Area, Wales | 7 | 3 | Pet / Breeding rats | unpublished |

**References for Supplementary Table 1**

- **Coleman, T.J. (2000)** The public health laboratory service (PHLS) and its role in the control of zoonotic disease. *Acta Trop*. Jul 21;**76**(1):71-5.
- **Dobly, A., Cochez, C., Goossens, E., De Bosschere, H., Hansen, P., Roels, S. & Heyman, P. (2012).** Sero-epidemiological study of the presence of hantaviruses in domestic dogs and cats from Belgium. *Res Vet Sci* **92**, 221-224.
- **Jameson, L. J., Logue, C. H., Atkinson, B., Baker, N., Galbraith, S. E., Carroll, M. W., Brooks, T. & Hewson, R. (2013a).** The continued emergence of hantaviruses: Isolation of a seoul virus implicated in human disease, United Kingdom, October 2012. *Eurosurveillance* **18**.
- **Jameson, L. J., Taori, S. K., Atkinson, B., Levick, P., Featherstone, C. A., van der Burgt, G., McCarthy, N., Hart, J., Osborne, J. C., Walsh, A. L., Brooks, T. & Hewson, R. (2013b).** Pet rats as a source of hantavirus in England and Wales, 2013. *Eurosurveillance* **18**.
- **Kudesia, G., Christie, P., Walker, E., Pinkerton, I., Lloyd, G. (1988)** Dual infection with leptospira and hantavirus. *Lancet*. Jun **18**;1(8599):1397.
- **Lloyd, G., Jones, N. (1986)** Infection of laboratory workers with hantavirus acquired from immunocytomas propagated in laboratory rats. *J Infect*. Mar;**12**(2):117-25
- **McKenna, P.; Clement, J.; Matthys, P.; Coyle, P.; McCaughey, C.** Serological evidence of Hantavirus disease in Northern Ireland. J. Med. Virol. 1994, 43, 33–38.
- **Pether, J.V., Jones N., Lloyd G. (1991) Acute hantavirus infection. Lancet. Oct 19;338(8773):1025.**
- **Pether, J.V., Lloyd, G. (1993)** The clinical spectrum of human hantavirus infection in Somerset, UK. *Epidemiol Infect*. Aug;**111**(1):171-5.
- **Phillips, M.J., Johnson, S.A., Thomson, R.K., Pether, J.V. (1991)** Further UK case of acute hantavirus infection. *Lancet*. Dec 14;**338**(8781):1530-1.
- **Rice, P., Kudesia, G., Leach, M**. **(1993)** Acute hantavirus infection. *J Infect.* Nov;**27**(3):342-4.
- **Walker, E., Boyd, A.J., Kudesia, G., Pinkerton, I.W. (1985)** A Scottish case of nephropathy due to Hantaan virus infection. *J Infect*. Jul;**11**(1):57-8.
- **Watson, A.R., Irving, W.L., Ansell, I.D. (1997)** Playing in a scrapyard and acute renal failure. *Lancet*. May 17;**349**(9063):1446.

**Supplementary Table 2** Histopathological findings for Cherwell Colony Rat Tissues

| **ID** | **Lung** | **Kidney** | **Liver** | **Brain** | **Salivary Gland** | **Spleen** | **Heart** | **Pancreas** | **Mand. ln.** | **Duodenum** | **Mammary** |
| --- | --- | --- | --- | --- | --- | --- | --- | --- | --- | --- | --- |
| 3776 | NAD | NAD | NAD | NAD | NAD | NAD | NAD | NAD | X | X | X |
| 3777 | NAD | NAD | NAD | NAD | NAD | NAD | NAD | X | X | X | X |
| 3778 | NAD | NAD | Vacuolar hepatopathy + | NAD | NAD | NAD | NAD | NAD | NAD | NAD | X |
| 3779 | NAD | NAD | NAD | NAD | NAD | NAD | NAD | X | X | X | X |
| 3780 | BALT hyperplasia +++ | NAD | NAD | NAD | NAD | NAD | NAD | X | X | X | X |
| 3781 | BALT hyperplasia ++  Interstitial pneumonia + | NAD | NAD | NAD | NAD | NAD | NAD | X | X | X | X |
| 3782 | BALT hyperplasia ++  Interstitial pneumonia + | NAD | NAD | NAD | NAD | NAD | NAD | X | X | X | X |
| 3783 | NAD | NAD | X | NAD | NAD | NAD | NAD | X | X | X | X |
| 3784 | BALT hyperplasia +  Emphysema + | Bowman’s capsule thickening ++  Interstitial nephritis and fibrosis +(#)  Tubular ectasia, attenuation and protein casts ++ | X | NAD | NAD | NAD | NAD | NAD | X | X | X |
| 3785 | BALT hyperplasia +  Interstitial pneumonia + | NAD | X | NAD | NAD | NAD | NAD | X | X | X | X |
| 3786 | BALT hyperplasia +++  Interstitial pneumonia + | NAD | NAD | NAD | NAD | NAD | NAD | X | X | X | X |
| 3787 | BALT hyperplasia +++  Interstitial pneumonia ++ | NAD | X | X | X | X | X | X | X | X | X |
| 3788 | BALT hyperplasia +  Interstitial pneumonia + | NAD | X | X | X | X | X | X | X | X | X |
| 3789 | BALT hyperplasia +++  Interstitial pneumonia + | Bowman’s capsule thickening +  Tubular ectasia, attenuation and protein casts + | X | X | X | X | X | X | X | X | Fibroadenoma |
| 3790 | BALT hyperplasia +  Interstitial pneumonia + | NAD | X | X | X | X | X | X | X | X | X |
| 3791 | BALT hyperplasia +++  Bronchiectasis +++ Bronchointerstitial pneumonia ++  Type II pneumocyte hyperplasia/trophy ++ | Bowman’s capsule thickening +  Tubular ectasia, attenuation, mineralisation and protein casts + | X | X | X | X | X | X | X | X | X |
| 3792 | BALT hyperplasia +  Bronchiectasis + | NAD | X | X | X | X | X | X | X | X | X |
| 3793 | BALT hyperplasia + | NAD | X | X | X | X | X | X | X | X | X |
| 3794 | BALT hyperplasia +++ | Interstitial nephritis +  Tubular ectasia, attenuation and protein casts + | X | X | X | X | X | X | X | X | X |
| 3795 | BALT hyperplasia + | NAD | X | X | X | X | X | X | X | X | X |
| 3796 | BALT hyperplasia +++  Bronchointerstitial pneumonia + | NAD | X | X | X | X | X | X | X | X | X |

“X” = tissue not sampled for that individual, NAD = no abnormalities detected, + = mild, ++ = moderate, +++ = marked

# The presence of increased connective tissue in the interstitum (fibrosis) was only a component of the histopathological changes observed in the kidney, so the inflammatory and degenerative changes, as well as urinary ectasis will account for an increase in size macroscopically


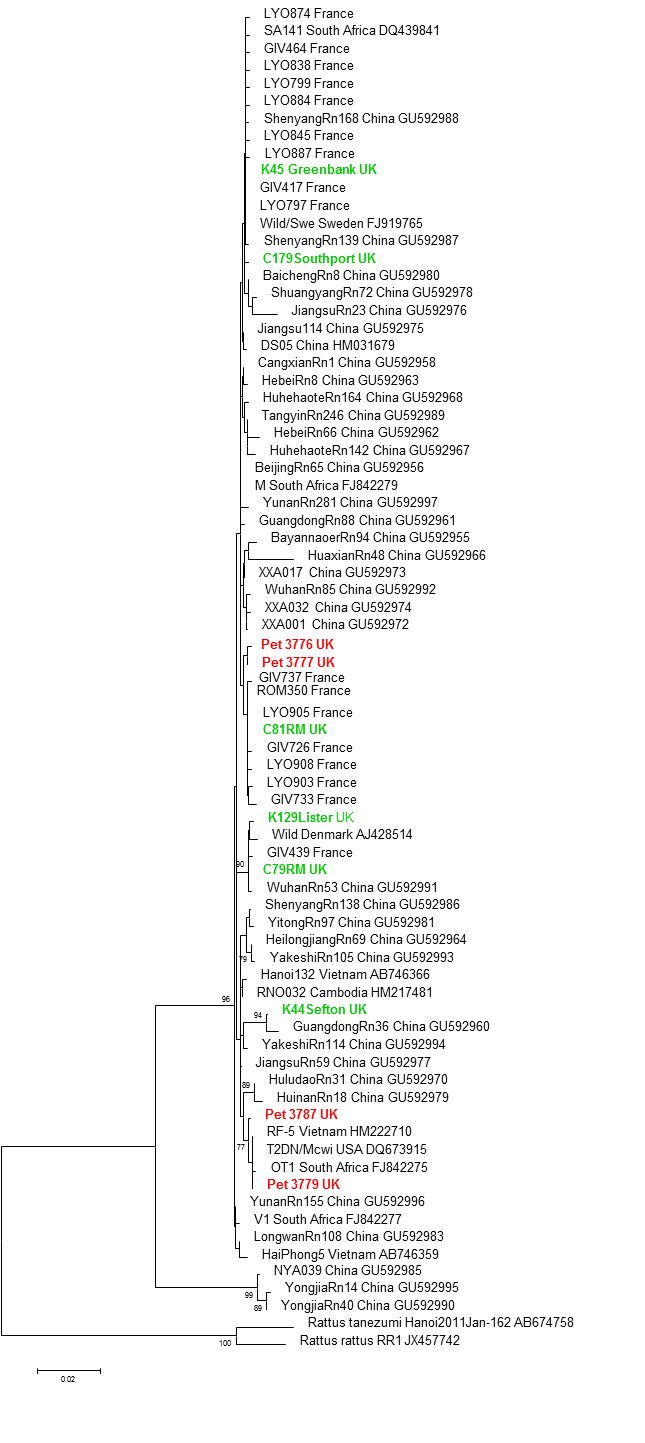


**Supplementary Figure 1.** Maximum likelihood phylogenetic tree (model HKY+Gamma) for partial *cyt b* segment sequences (833 nt) n=77 in the MEGA6 package of software with bootstrap of 10,000 . Only bootstrap support of >70 % are shown. The phylogenetic position of the UK pet rats (green = wild, pet = red) are shown in relation to representative *R. norvegicus* sequences. Genbank accession numbers are shown next to taxa names. Pairwise comparisons among all sequences (including out groups) ranged from 0 to 12 %. All pet rat cyt b sequences in this study had an average genetic distance of 0.5 % (ranging between 0.1 to 0.8 %) and compared to wild UK rats an average genetic distance of 0.8 % (ranging between 0.4 to 1.3 %). Compared to Norway rats worldwide (excluding individuals from mountainous areas of China; Lin et al., 2012) the pet rat sequences had an average distance of 0.7 % (range between 0 to 2.0 %). The average genetic distance to the outgroup *R. tanezumi* and *R. rattus* was 11.1 % (ranging from 10.7 to 12 %).

**Supplementary Data**

Hantavirus FRNT Assays

Briefly, sera were serially diluted four-fold and mixed with an equal volume containing 30–70 focus forming units (FFU) of virus per 100 ml. The mixtures were incubated for 1 hr and 200µl subsequently inoculated into the wells of six-well tissue plates containing confluent Vero E6 cell monolayers. After adsorption for 1 hr, the wells were overlaid with 3 ml of a mixture of 1 part agarose and 1 part 2 x basal Eagle’s medium supplemented with antibiotics. Plates were incubated at 37^o^C for 6 days for the HTNV (76-118), 9 days for SEOV (80-39) assays or 8 days for the DOBV (Slovenia) and PUUV (Kazaan) assays. Virus-infected cells were detected with hantavirus-specific polyclonal antisera, followed by peroxidase-labelled goat antibodies and substrate.
